# Prognostic value of tripartite motif (TRIM) family gene signature from bronchoalveolar lavage cells in idiopathic pulmonary fibrosis

**DOI:** 10.1186/s12890-022-02269-4

**Published:** 2022-12-06

**Authors:** Mi Zhou, Jie Ouyang, Guoqing Zhang, Xin Zhu

**Affiliations:** 1 Department of Respiratory and Critical Care Medicine, The First Affiliated Hospital of Chongqing Medical University, Chongqing, China; 2 Department of Urology, The First Affiliated Hospital of Chongqing Medical University, No. 1 Youyi Road, Yuzhong District, Chongqing, 400016 China

**Keywords:** Idiopathic pulmonary fibrosis, Tripartite motif, Overall survival, Risk signature, Prognosis

## Abstract

**Background:**

Tripartite motif (TRIM) family genes get involved in the pathogenesis and development of various biological processes; however, the prognostic value of TRIM genes for idiopathic pulmonary fibrosis (IPF) needs to be explored.

**Methods:**

We acquired gene expression based on bronchoalveolar lavage (BAL) cells and clinical data of three independent IPF cohorts in the GSE70866 dataset from the Gene expression omnibus (GEO) database. Differentially expressed TRIM genes (DETGs) between IPF patients and healthy donors were identified and used to establish a risk signature by univariate and multivariate Cox regression analysis in the training cohort. The risk signature was further validated in other IPF cohorts, and compared with previously published signatures. Moreover, we performed functional enrichment analysis to explore the potential mechanisms. Eventually, the quantitative real time PCR was conducted to validate the expressions of the key genes in BAL from 12 IPF patients and 12 non-IPF controls from our institution.

**Results:**

We identified 4 DETGs including TRIM7, MEFV, TRIM45 and TRIM47 significantly associated with overall survival (OS) of IPF patients (*P* < 0.05). A multiple stepwise Cox regression analysis was performed to construct a 4-TRIM-gene prognostic signature. We categorized IPF patients into one low-risk group and the other high-risk group as per the average risk value of the TRIM prognostic signature in the training and validation cohorts. The IPF individuals in the low-risk group demonstrated an obvious OS advantage compared with the high-risk one (*P* < 0.01). The time-dependent receiver operating characteristic approach facilitated the verification of the predictive value of the TRIM prognostic signature in the training and validation cohorts, compared with other published signatures. A further investigation of immune cells and IPF survival displayed that higher proportion of resting memory CD4+ T cells and resting mast cells harbored OS advantage over lower proportion, however lower proportion of neutrophils, activated dendritic cells and activated NK cells indicated worse prognosis.

**Conclusion:**

The TRIM family genes are significant for the prognosis of IPF and our signature could serve as a robust model to predict OS.

## Background

Idiopathic pulmonary fibrosis (IPF) is a chronic and lethal interstitial lung disease, characterized by excessive extracellular matrix protein deposition and irreversible lung damage, resulting in progressive ventilation dysfunction, hypoxemia, respiratory failure and death [1, 2]. The underlying pathophysiological mechanism of IPF is complex, and the current favored hypothesis proposes that IPF arises as a result of repetitive injury to the alveolar epithelium in genetically susceptible and aged individuals, indicating the significant value of exploring genetic and epigenetic information of alveolar epithelium in IPF [3, 4]. Nintedanib and pirfenidone are two antifibrotic medications approved for slowing the rate of lung function decline in IPF [5]. An observational study found that, if untreated with antifibrotics, IPF patients are predicted of a 50% all-cause mortality at 3 years, which represent as the natural course of the disease [6]. Therefore, there is an urgent requirement for reliable biomarkers for early diagnosis, monitoring disease progression and prediction of a patient’s response to medical antifibrotic treatment.

Alterations in genetic and molecular information obtained from lung tissues, peripheral blood mononuclear cells, bronchoalveolar lavage (BAL) cells, were found to be associated with disease severity and mortality of IPF patients [7]. Recently, increasing evidence reveals that tripartite motif (TRIM) family members widely involve in various biological processes, including cell proliferation, differentiation, apoptosis, tumorigenesis, and innate immunity [8, 9]. With regard to specific molecular structure, most of TRIM proteins possess E3-ubiquitin ligase activity and are characterized with the presence of a RING finger domain, followed by one or two B-box domains and a coiled-coil domain (CCD) in the amino terminal region [10, 11].

In this study, we systematically compared TRIM gene expressions of BAL cells between normal individuals and IPF cases, and differentially expressed TRIM genes (DETGs) associated with IPF prognosis were used to construct a prognostic signature in a training cohort and further validated by other two independent cohorts. The potential mechanism was further investigated between low-risk and high-risk groups based on the prognostic signature.

## Methods

### Data collection

Gene expression profile based on BAL cells and clinical data of GSE70866 was obtained from the Gene Expression Omnibus (GEO) database (https://www.ncbi.nlm.nih.gov/geo/), which included a cohort of 62 IPF patients from Freiburg, Germany and a cohort of 50 IPF patients from Siena, Italy based on GPL14550 platform (Agilent-028004 SurePrint G3 Human GE 8 × 60 K Microarray), and another cohort consisting of 64 patients from Leuven, Belgium based on GPL17077 platform (Agilent-039494 SurePrint G3 Human GE v2 8 × 60 K Microarray) [12]. A total of 95 TRIM gene family members were obtained HUGO Gene Nomenclature Committee (HGNC) at the European Bioinformatics Institute (https://www.genenames.org/) (Table 1).

**Table 1:** Tripartite motif family genes

MID2	TRIM21	TRIM40	TRIM51CP	TRIM63
TRIM2	TRIM22	TRIM41	TRIM51DP	TRIM64
TRIM3	TRIM23	TRIM42	TRIM51EP	TRIM64B
TRIM4	TRIM24	TRIM43	TRIM51FP	TRIM64C
TRIM5	TRIM25	TRIM43B	TRIM51G	TRIM64DP
TRIM6	TRIM26	TRIM43CP	TRIM51HP	TRIM64EP
TRIM7	TRIM26BP	TRIM44	TRIM51JP	TRIM64FP
TRIM8	TRIM27	TRIM45	TRIM52	TRIM65
TRIM9	TRIM28	TRIM46	TRIM53AP	TRIM66
TRIM10	TRIM29	TRIM47	TRIM53BP	TRIM67
TRIM11	TRIM31	TRIM48	TRIM53CP	TRIM68
TRIM13	TRIM32	TRIM49	TRIM54	TRIM69
TRIM14	TRIM33	TRIM49B	TRIM55	TRIM71
TRIM15	TRIM34	TRIM49C	TRIM56	TRIM72
TRIM16	TRIM35	TRIM49D1	TRIM58	TRIM73
TRIM17	TRIM36	TRIM49D2	TRIM59	TRIM74
MID1	TRIM37	TRIM50	TRIM60	TRIM75
PML	TRIM38	TRIM51	TRIM61	CMYA5
MEFV	TRIM39	TRIM51BP	TRIM62	TRIM77

### Exploration of DETGs

DETGs between 62 IPF patients and 20 healthy donors from the Freiburg cohort were identified using the “limma” package on R software, at the threshold of [log2FoldChange (log2FC)] > 0 and a false discovery rate (FDR) < 0.2 [13]. Volcano plots and DETGs heatmap analyses were done using “ggplot2” and “pheatmap” package, respectively.

### Construction and validation of a prognostic DETG signature

To construct a reliable prognostic signature based on the DETGs, the Freiburg cohort was used as the training cohort to screen for hub genes, and the other two independent cohorts (Siena and Leuven cohorts) were used for validation. Univariate Cox regression analysis was used to select DETGs associated with overall survival (OS) of IPF patients at the threshold of *P* < 0.05. OS-related DETGs were visualized on forest maps and applied to establish prognostic signature by stepwise multivariate Cox regression analysis. In the training cohort, the signature for prediction was to multiply the expression level of each selected prognostic gene by its corresponding relative regression coefficient weight as follows:

$$\mathrm{Risk score}={\sum }_{k=1}^{n}coefficient\left(k\right)*gene(k)$$

(n represents the total number of genes). The risk score of each patient in the training cohort was calculated with the signature and the median risk score was used to classify the total cohort into high-risk group with a higher risk score than the median, and low-risk group with a lower risk score than the median. The time-dependent prognostic value of the gene signature was investigated using the Kaplan–Meier curve and log-rank test was used to compare the survival difference between the above-mentioned high- and low-risk groups [14]. Receiver operating characteristic (ROC) curves of the two groups were plotted to evaluate the sensitivity and specificity of the signature we established [15, 16]. Consistent with the training set, the patients in the validation datasets including Siena and Leuven cohorts were classified into the high- and low-score groups by comparing the risk score of each patient with the calculated median risk score from each cohort.

### Construction of a prognostic nomogram

Univariate and multivariate Cox regression analyses were applied to determine independent clinical factors, including age, gender, GAP and risk-score. Through “rms” and “survival” packages in R, a prognostic nomogram was established to evaluate the probability of OS in IPF patients in the training cohort.

### Gene set enrichment analysis

Gene set enrichment analysis (GSEA) version 4.1.0 was used to explore potential molecular mechanisms between patients in low- and high-risk groups underlying our constructed signature [17]. The Kyoto Encyclopedia of Genes and Genomes (KEGG) gene sets in C2 were used to explore enriched terms and the cut-off values for the significance of outcomes were *P* < 0.05, and |normalized enrichment score (NES) |> 1 [18].

### Evaluation of immune cell fractions

Relative proportions of 22 given kinds of immunocytes in IPF samples were assessed by applying the CIBERSORT deconvolution algorithm [19, 20]. Through this method, estimated abundances of immunocytes had been assessed by 22 given kinds of immunocytes accompanying with 100 permutations.

### BAL collection and quantitative real-time PCR

We obtained BAL from 12 IPF patients and 12 non-IPF controls who underwent bronchoscopy in the First Affiliated Hospital of Chongqing Medical University. Written informed consent was obtained from all the patients, and the research was approved by the Ethical Committee of our institution. The BAL samples were immediately isolated and 1 ml Trizol to was added to the cell samples,then snap-frozen in liquid nitrogen and preserved in a − 80 °C freezer. Total RNA was isolated from frozen samples using the TRUEscript RT MasterMix (DF Biotech., CHENGDU, China) according to manufacturer’s instructions. Then total RNA was reverse-transcribed into complementary DNA using the TransScript First-Strand cDNA Synthesis kit (AiDLAB Biotech, Beijing, P.R. China). Quantitative real-time PCR (qRT-PCR) was then performed using the SYBR Green QPCR Mix (DF Biotech., CHENGDU, China). The data was calculated through the 2^−ΔΔCt^ strategy, normalizing with GAPDH. The primer sequences used for qRT-PCR in this study are listed in Table 2.

**Table 2:** Sequences of hub gene primers for qRT-PCR

Gene	Forward primer(5′–3′)	Reverse primer(5′–3′)
TRIM7	CCAACCACAGTCTCTTCT	GAACTTCTTCAGCATCCC
MEFV	GGAGGTTGGAGACAAGACA	CCACCCAGTAGCCATTCT
TRIM45	GGAGACTCTGACACAAGC	CACCTGAGCATCACATACA
TRIM47	TCTTCACCCACAGACTCA	TCCTCTTCAGCACGGATA
GAPDH	TGCACCACCAACTGCTTAGC	GGCATGGACTGTGGTCATGAG

### Statistical analysis

R software (version 4.1.2) packages were used to analyze GEO data. The qRT-PCR data was analyzed using unpaired Student’s t-test and visualized by using GraphPad Prism 9.4.1 (GraphPad Software, LLC). *P* < 0.05 was statistically significant.

## Results

### Identification of OS-related DETGs

**Fig. 1:**
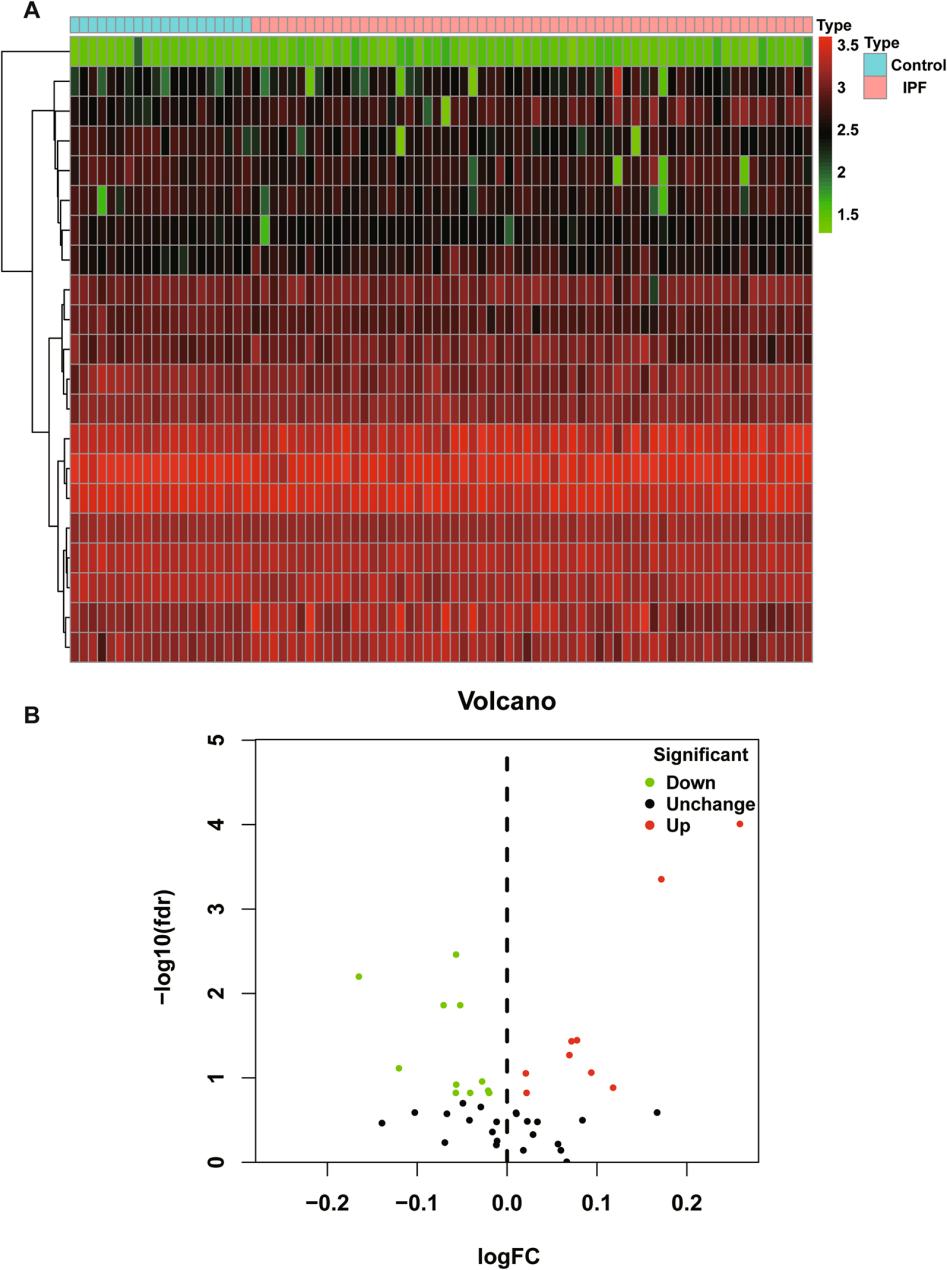
**A** Heatmap indicated 21 DETGs between 62 IPF patients and 20 healthy donors from the Freiburg cohort. **B** Volcano plot represented upregulated and down-regulated DETGs in the Freiburg cohort

**Fig. 2:**
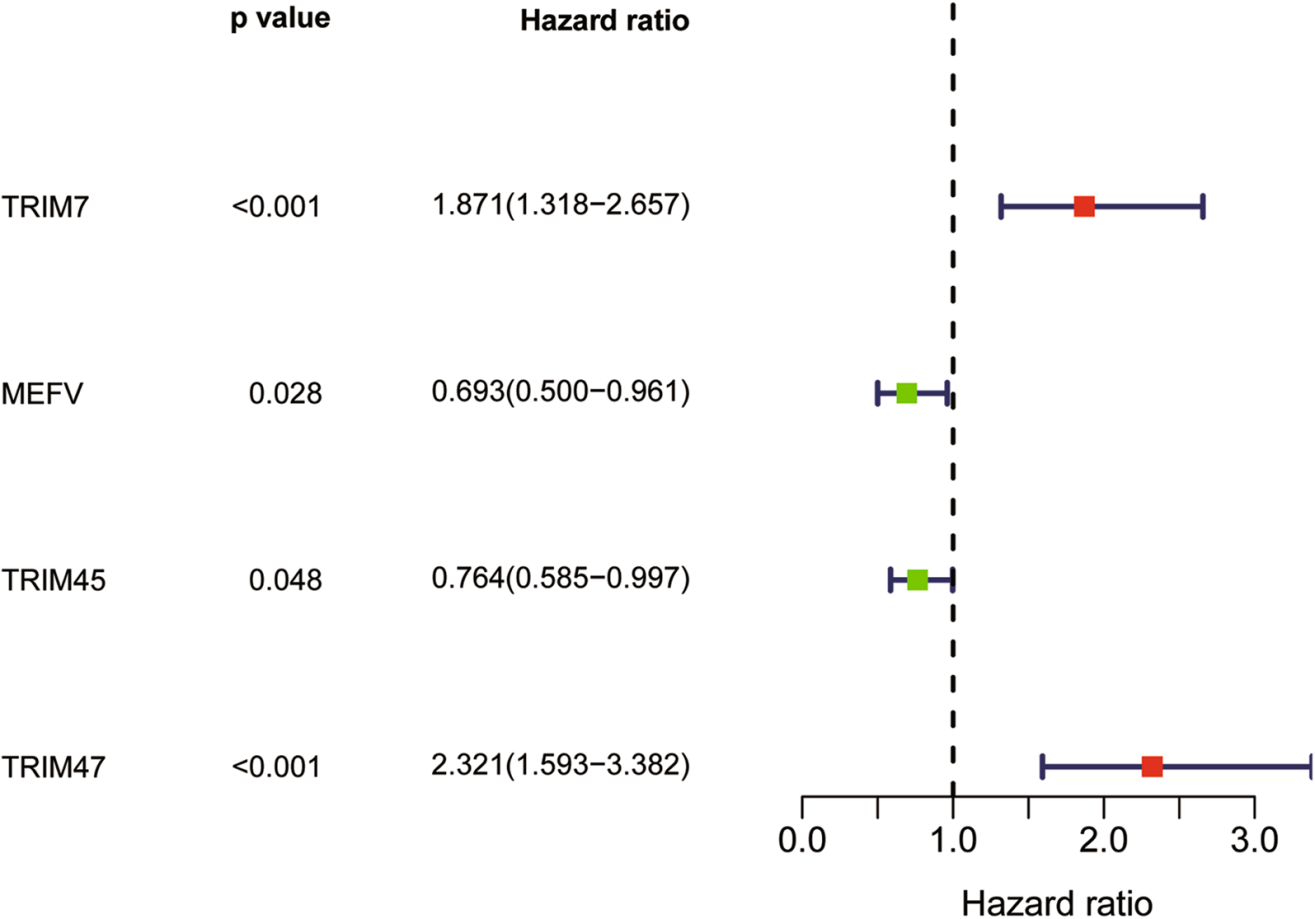
Forest plot of univariate Cox regression analysis of prognostic DETGs in the Freiburg cohort

Among all the TRIM family members, 21 DETGs were obtained between 62 IPF patients and 20 healthy donors from the Freiburg cohort, as demonstrated in the heat map (Fig. 1A) and volcano plot (Fig. 1B). The prognostic significance of the 21 DETGs was explored using univariate Cox regression. As a result, only four OS-related DETGs including TRIM7, MEFV, TRIM45 and TRIM47 were obtained as demonstrated in the forest plot (Fig. 2). Among them, TRIM7 and TRIM47 may be regarded as risky genes for OS of IPF patients, whereas MEFV and TRIM45 may be protective genes.

### Construction of the TRIM prognostic signature

**Fig. 3:**
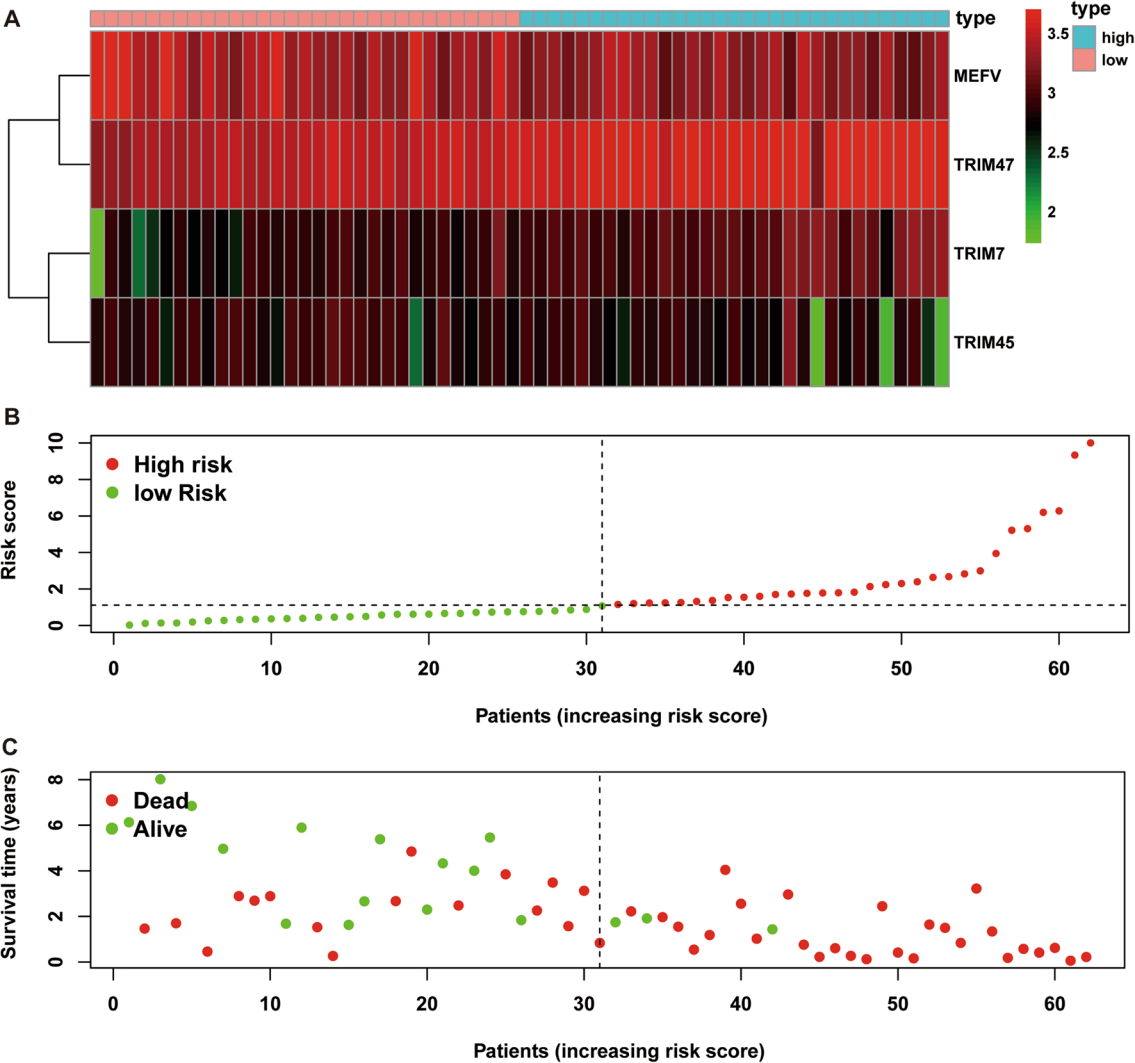
The heatmap of mRNA expression of the four-gene signature (**A**), risk scores and distributions (**B**), and survival time and survival status of IPF patients (**C**) in the Freiburg cohort

**Fig. 4:**
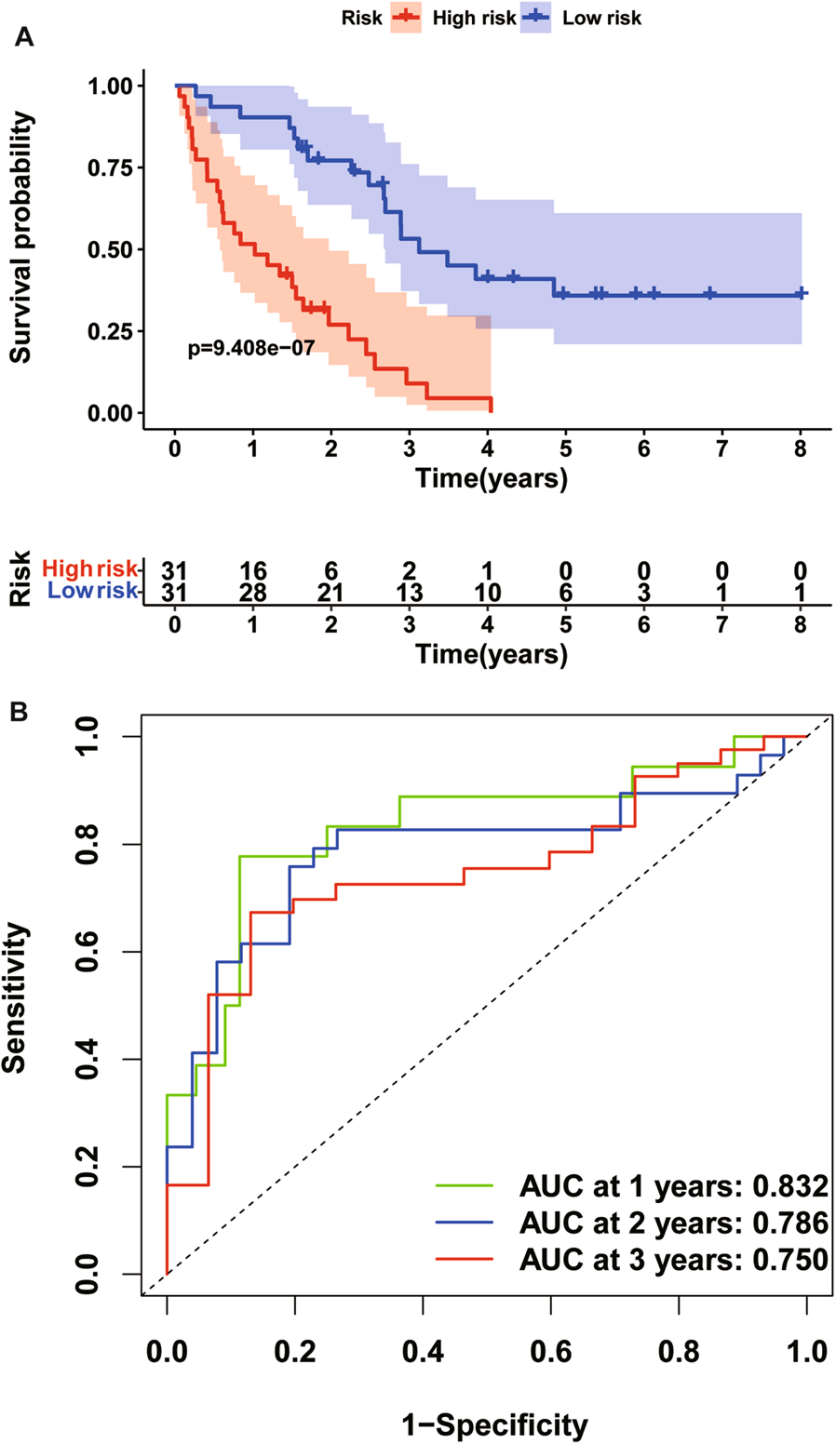
Time-dependent ROC analysis (**A**) and Kaplan–Meier curve of the four-gene signature in the Freiburg cohort (**B**). ROC receiver operating characteristic

A multiple stepwise Cox regression analysis was performed to investigate prognostic significance of the four OS-related DETGs, which were further selected to construct the prognostic signature in IPF patients. The risk score for each patient was calculated based on the following formula: risk score = (0.431) × TRIM7 + (− 0.304) × MEFV + (− 0.409) × TRIM45 + (0.553) × TRIM47 (Table 3). The coefficients of these genes indicated their impact on survival prediction. We then divided IPF patients into the low-risk group (n = 31) and high-risk group (n = 31) according to the median risk score as the cut-off point. The patients’ gene expression levels, risk scores, survival time and survival status were demonstrated in Fig. 3A–C. The K-M results indicated that the OS rate of IPF patients in the low-risk group was significantly higher than that in the high-risk group (*P* < 0.001, Fig. 4A). Moreover, the time-dependent ROC curve showed that the area under the ROC curve (AUC) of this prognostic signature at 1, 2, and 3 years were 0.832, 0.786 and 0.750, respectively (Fig. 4B), with moderate predictive performance.

**Table 3:** The selected genes for prognostic signature in IPF patients

Gene	Description	Coef	HR (95%CI)	*P* value
TRIM7	Tripartite motif containing 7	0.431	1.539 (1.053,2.250)	0.026
MEFV	MEFV innate immuity regulator, pyrin	− 0.304	0.738 (0.497, 1.097)	0.133
TRIM45	Tripartite motif containing 45	− 0.409	0.664 (0.492, 0.896)	0.007
TRIM47	Tripartite motif containing 47	0.553	1.739 (1.219, 2.481)	0.002

coef, coefficients; HR, hazard ratio; 95% CI, 95% confidence interval

### Validation of the TRIM prognostic signature

**Fig. 5:**
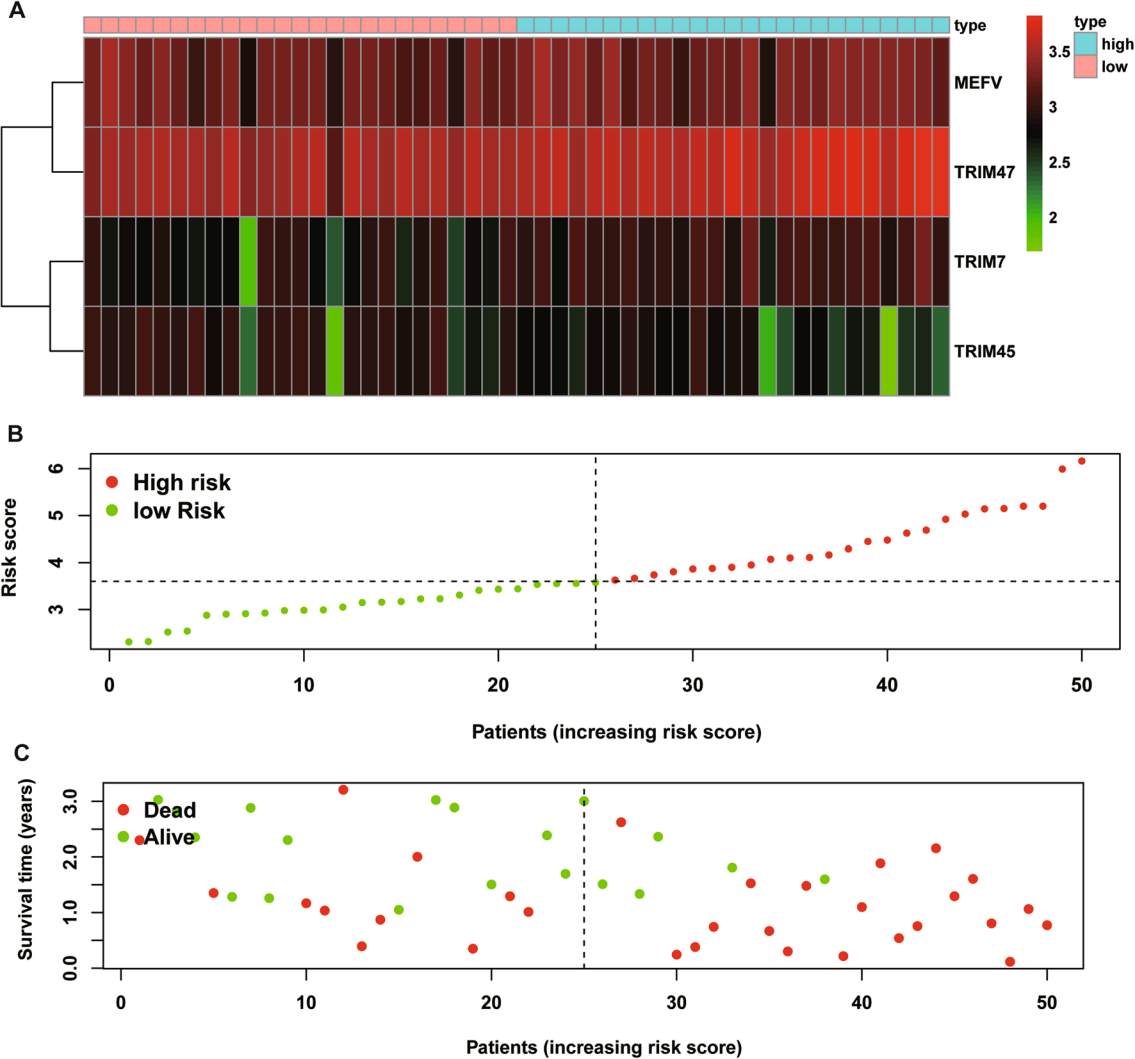
The heatmap of mRNA expression of the four-gene signature (**A**), risk scores and distributions (**B**), and survival time and survival status of IPF patients (**C**) in the Siena cohort

**Fig. 6:**
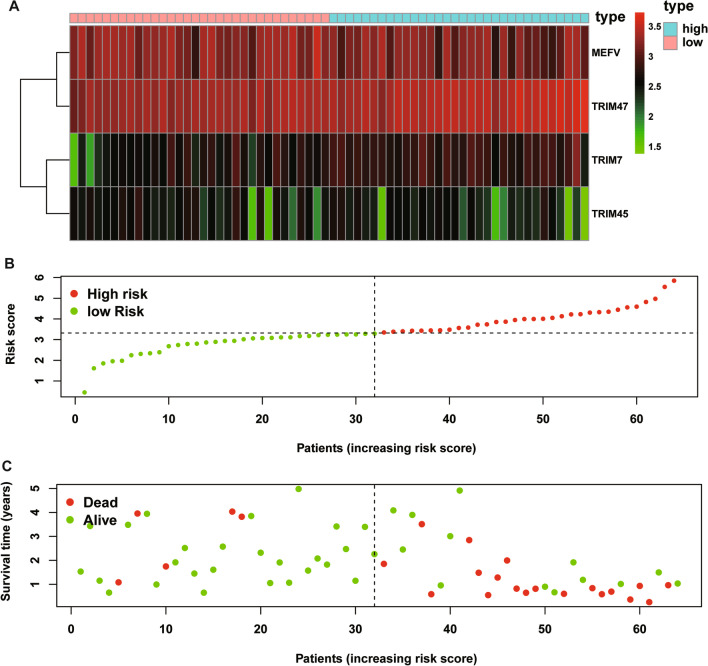
The heatmap of mRNA expression of the four-gene signature (**A**), risk scores and distributions (**B**), and survival time and survival status of IPF patients (**C**) in the Leuven cohort

**Fig. 7:**
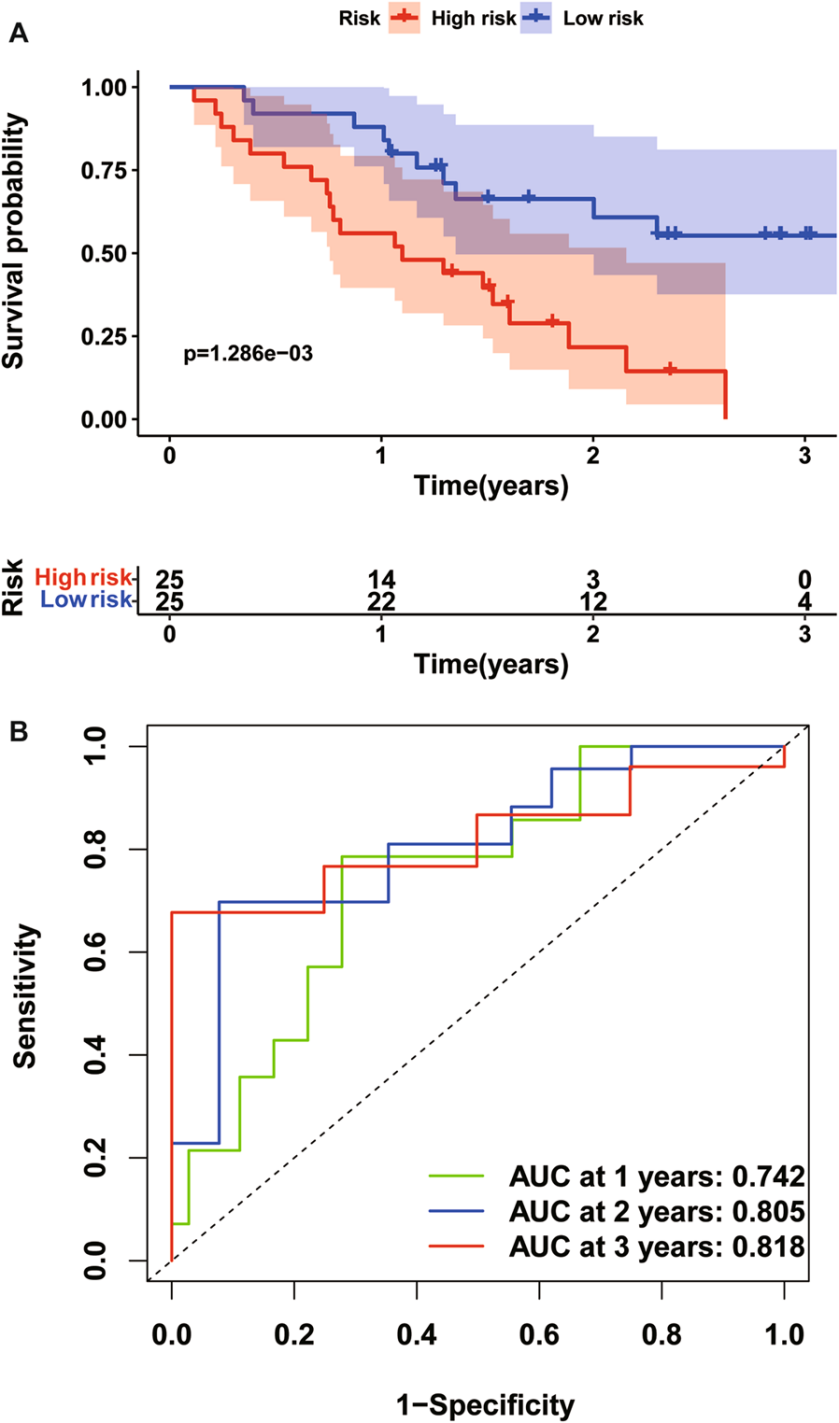
Time-dependent ROC analysis (**A**) and Kaplan–Meier curve of the four-gene signature in the Siena cohort (**B**). ROC receiver operating characteristic

**Fig. 8:**
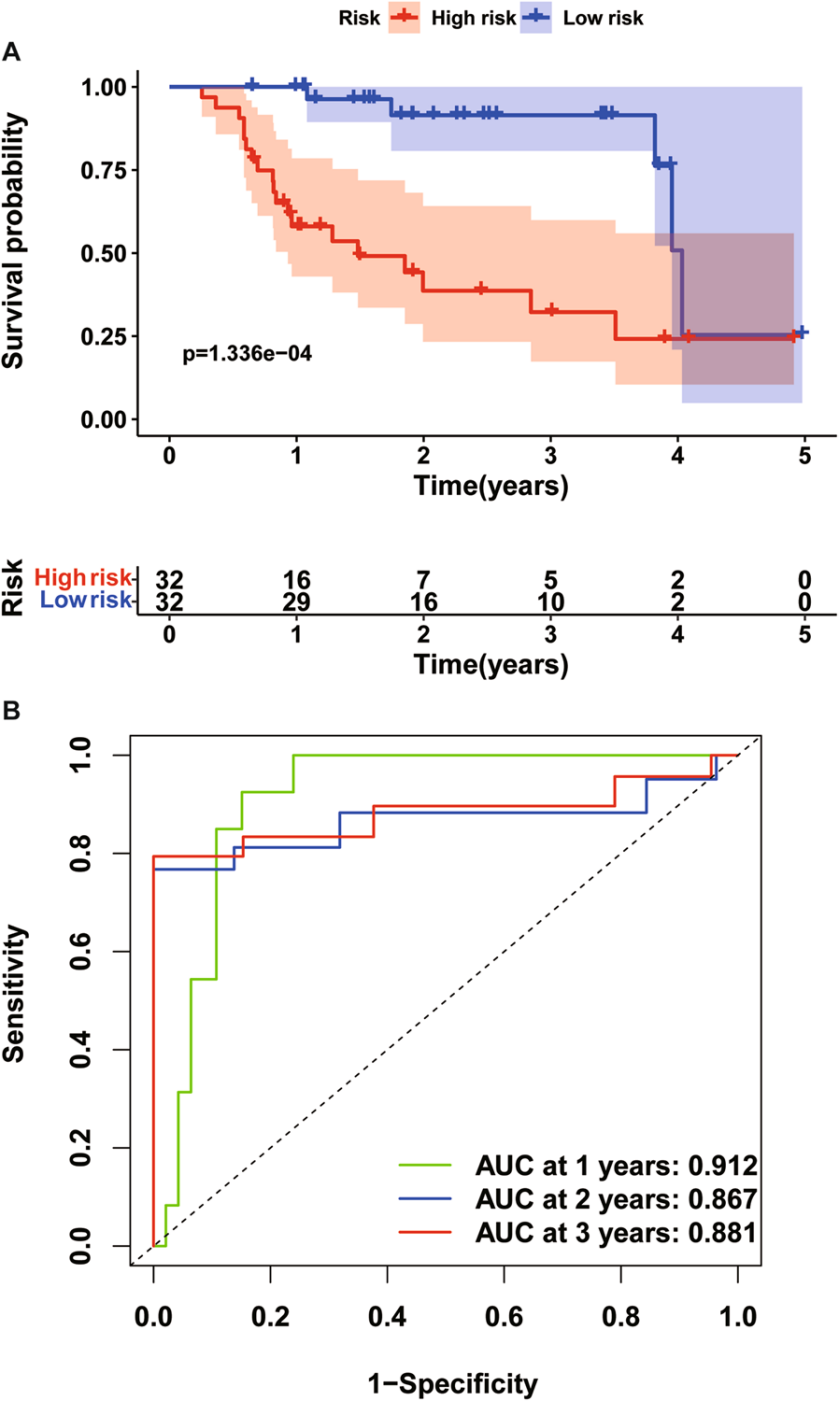
Time-dependent ROC analysis (**A**) and Kaplan–Meier curve of the four-gene signature in the Leuven cohort (**B**). ROC receiver operating characteristic

**Fig. 9:**
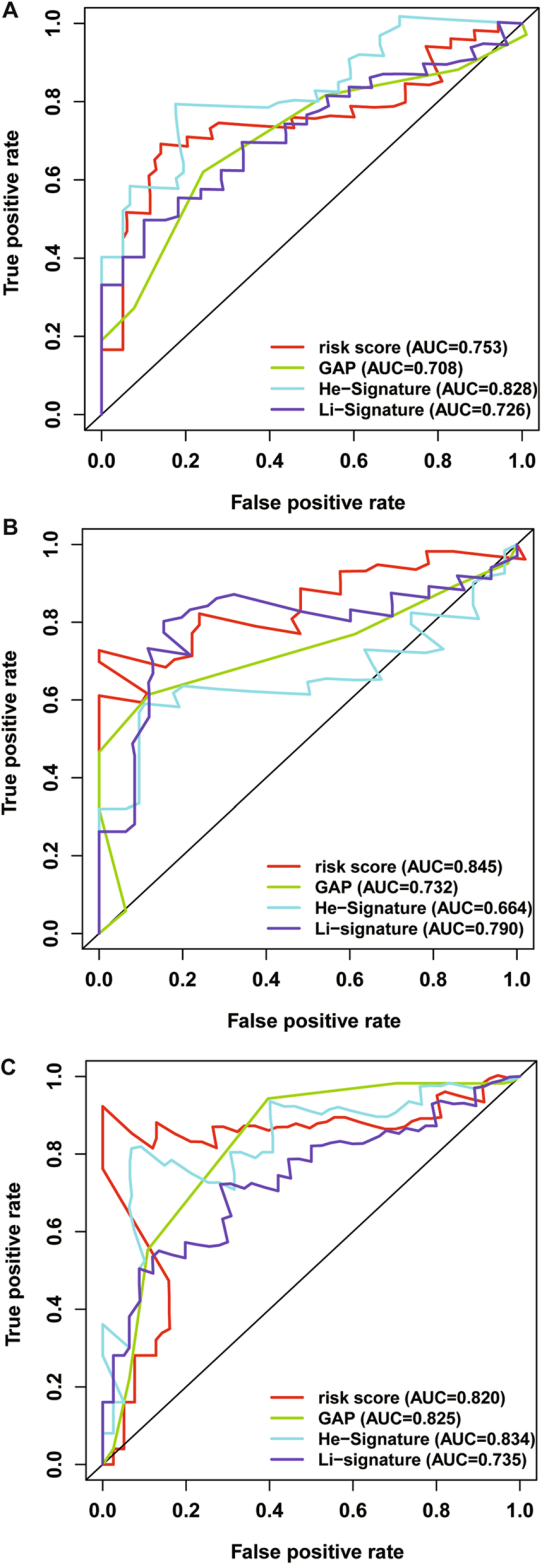
Time-dependent ROC analysis to compare predictive value of our signature with GAP, He’s signature and Li’s signature in the Freiburg cohort (**A**), the Siena cohort (**B**) and the Leuven cohort (**C**)

To verify the predictive performance of the TRIM prognostic signature in other IPF cohorts, we conducted similar analyses in other two independent cohorts. Gene expressions, risk scores, survival time and survival status of Siena cohort and Leuven cohort were demonstrated in Figs. 5A–C and 6A–C. In the Siena cohort, the OS of IPF patients in the high-risk group was significantly worse than that of patients in the low-risk group (*P* < 0.01) (Fig. 7A), with AUC values of 0.742, 0.805, and 0.818 at 1, 2, and 3 years, respectively (Fig. 7B). Interestingly, the OS rate between low- and high-risk groups was also significantly different (Fig. 8A), with AUC values of 0.912, 0.867, and 0.881 at 1, 2, and 3 years, respectively (Fig. 8B). In conclusion, this signature also demonstrated good performance for both independent validation cohorts. Compared with GAP and other signatures, our signature showed superior predictive value over GAP and comparable value with He’s signature and Li’s signature in the Freiburg cohort (Fig. 9A), the Siena cohort (Fig. 9B) and the Leuven cohort (Fig. 9C).

### Independent clinical factors

**Fig. 10:**
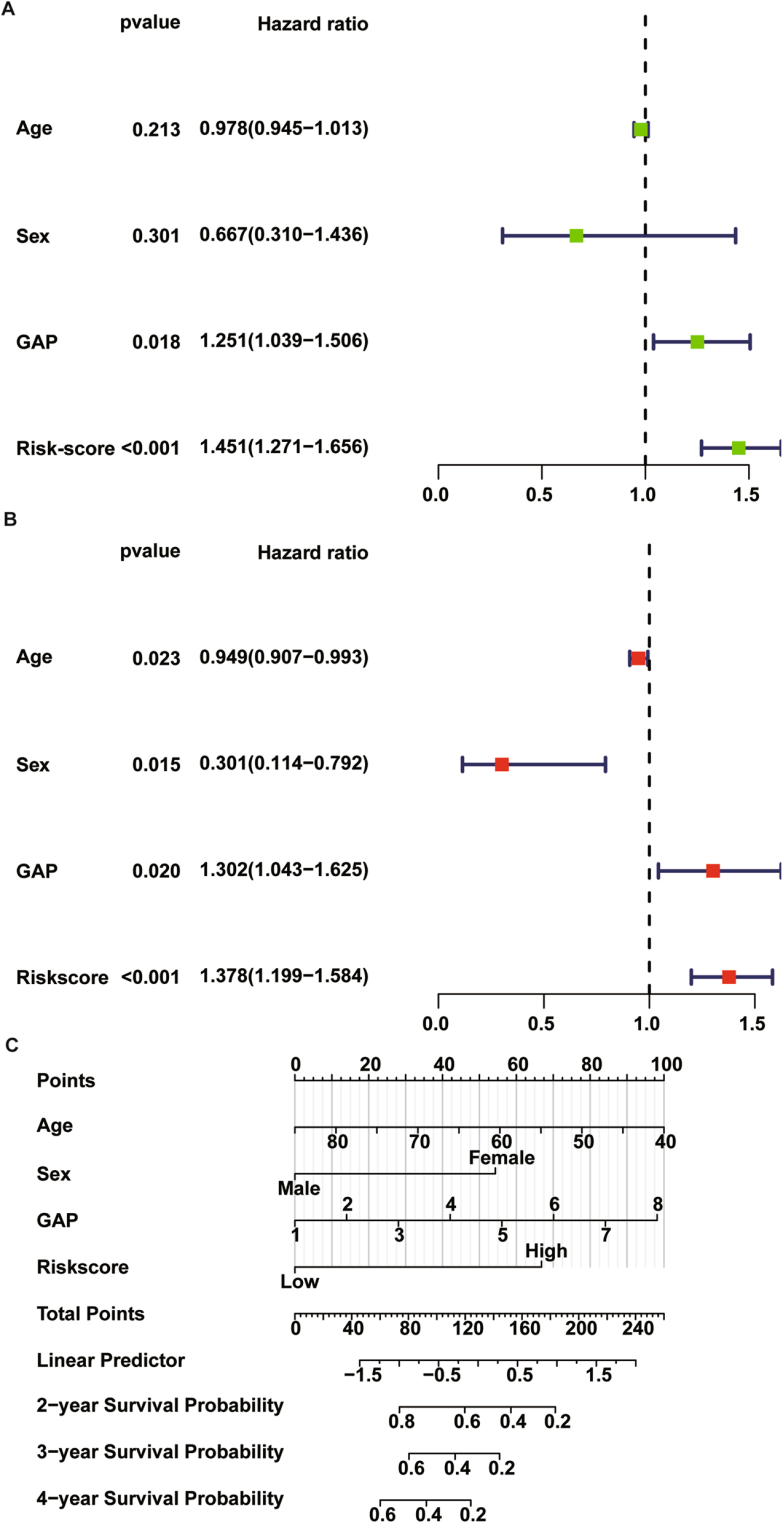
Univariate Cox regression (**A**) and multivariate Cox regression (**B**) analyses were performed using clinical data and TRIM prognostic signature to assess the independent prognostic factors in IPF samples; (**C**) a nomogram consisted of risk score and other clinical parameters including age, sex and GAP

Univariate and multivariate Cox regression analyses were performed using clinical data and TRIM prognostic signature to assess the independent prognostic factors in IPF samples. Both univariate and multivariate Cox regression analysis confirmed that GAP (HR = 1.302, 95% CI [1.043–1.625]; *P* = 0.02), risk score (HR = 1.378, 95% CI [1.199–1.584]; *P* < 0.001) were significant independent risk factors (Fig. 10A, B). Based on the results, the risk score can be used as an independent prognostic factor without being affected by clinicopathological features. A nomogram consisting of age, sex, GAP and risk score was constructed to predict prognosis of IPF patients as shown in Fig. 10C.

### Signaling pathways analysis of high-risk group

**Fig. 11:**
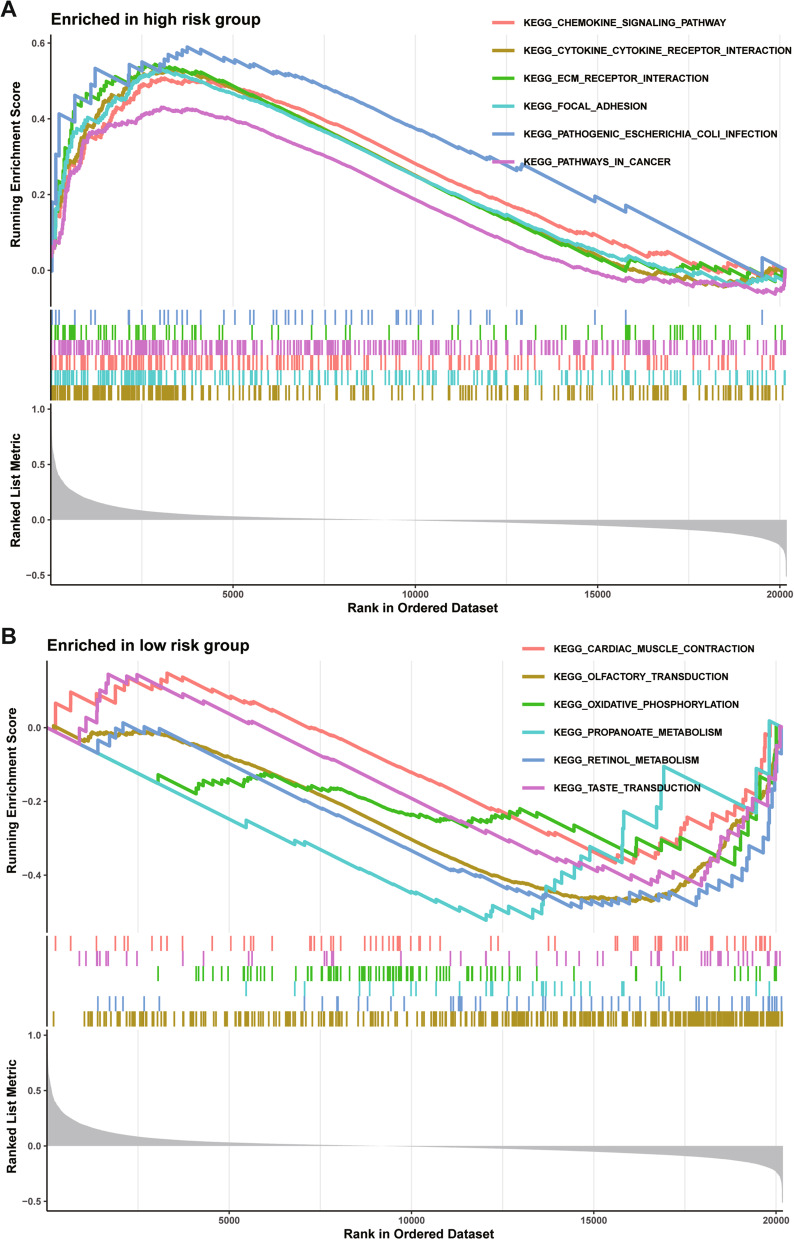
The KEGG functional enrichment in the high-risk group (**A**) and low-risk group (**B**) by GSEA method in the Freiburg cohort

In our study, patients in the high-risk group exhibited worse survival. We used GSEA to investigate the potentially important pathways causing different prognoses in the two groups. A KEGG functional enrichment analysis showed that chemokine signaling pathway, cytokine-cytokine receptor interaction, ECM-receptor interaction, focal adhesion, pathogenic escherichia coli infection, pathways in cancer (Fig. 11A). Cardiac muscle contraction, olfactory transduction, oxidative phosphorylation, propanoate metabolism, retinol metabolism and taste transduction (Fig. 11B).

### Profile of immune cell subtype distribution pattern

**Fig. 12:**
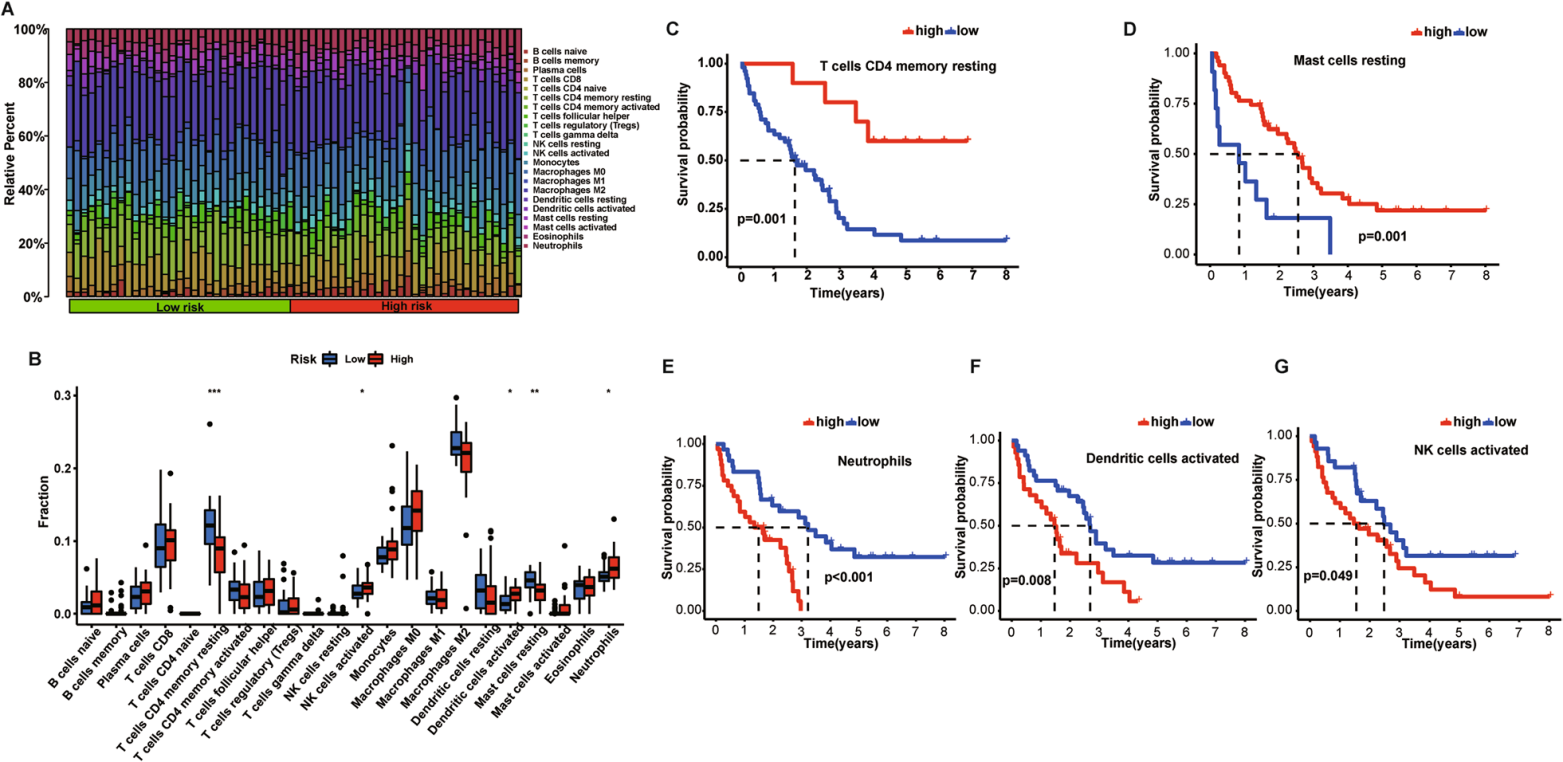
Histogram of distributions of various immune cells in each sample from low- and high-risk groups (**A**); comparison of different types of immune cells between low- and high-risk groups (**B**); Kaplan–Meier curve between low and high proportion groups of CD4 + T cells (**C**), resting mast cells (**D**), neutrophils (**E**), activated dendritic cells (**F**) and activated NK cells (**G**)

As demonstrated in Fig. 12A, the histogram shows distribution of various immune cells in each sample from low- and high-risk groups. Different colors represent different types of immune cells. Among the 22 immune cells, resting memory CD4+ T cells (*P* < 0.001), resting mast cells (*P* < 0.01), neutrophils (*P* < 0.05), activated dendritic cells (*P* < 0.05) and activated natural killer (NK) cells (*P* < 0.05) were significantly different between low- and high-risk groups (Fig. 12B). Then we conducted a further investigation of the five immune cells and IPF survival, and found that higher proportion of resting memory CD4+ T cells and resting mast cells harbored OS advantage over lower proportion, which were also consist with their higher fractions in low-risk group (Fig. 12C, D). Moreover, lower proportion of neutrophils, activated dendritic cells, activated NK cells indicated worse prognosis, consist with their lower fractions in low-risk group than high-risk group (Fig. 12E–G).

### Validation of expression levels of key genes in the clinical samples

**Fig. 13:**
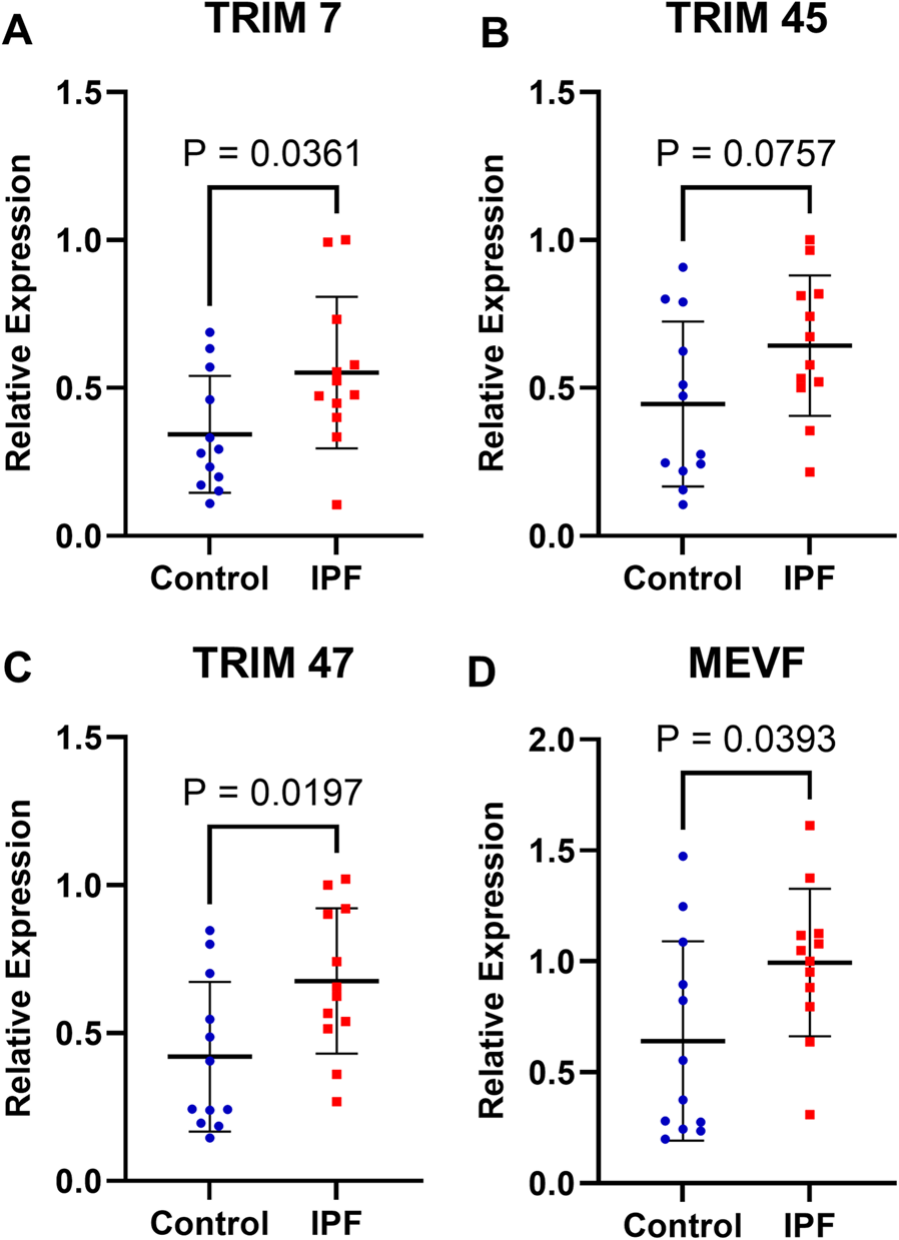
The mRNA expression of four genes in BAL from 12 IPF patients and 12 non-IPF controls **A** the mRNA expression of TRIM 7; **B** the mRNA expression of TRIM 45; **C** the mRNA expression of TRIM 47; **D** the mRNA expression of MEVF

To confirm the previous results, we detected the expression levels of the four DETGs including TRIM7, MEFV, TRIM45 and TRIM47, by qRT-PCR. It showed a higher mRNA expression trend of TRIM7, TRIM47 and MEFV in BAL cells of IPF patients, compared with non-IPF controls (Fig. 13A, C, D), consistent with previous results. However, the mRNA expression of TRIM45 between IPF and control groups was not significantly different (Fig. 13B).

## Discussion

Idiopathic pulmonary fibrosis (IPF) is a fatal condition, the most lethal form of interstitial pneumonia characterized with irreversible fibrosis in pulmonary parenchyma with unspecific etiology, leading to uncontrollable infection, respiratory failure and even death [21]. Increasing evidence suggested different clinical phenotypes of IPF resulted in variable disease courses over time [22]. With respect to etiology, genetic and environmental factors might cause repetitive local microinjuries to aging alveolar epithelium, which further triggers aberrant epithelial–fibroblast communication, induction of matrix-producing myofibroblasts, remodeling of the interstitium, and dysregulated repair of the injured lung [23, 24].

A multidimensional prognostic staging system GAP model, consisting of gender (G), age (A), and 2 lung physiology (P) variables including forced vital capacity (FVC) and diffusion capacity of the lung for carbon monoxide (DLCO), was firstly constructed and validated in 2012, with good predictive and discriminative performance in the prognosis of IPF [25]. A modified GAP model with predicted vital capacity (VC) used as a GAP variable instead of FVC, was further investigated and validated in the Japanese validation and Korean national cohorts [26]. Intriguingly, an Italian-developed TORVAN score based on data from two independent cohorts demonstrated significant improvement of discriminative performance in prediction of risk of death compared to GAP model by adding comorbidity variables [27]. Besides clinical predictive parameters, because molecular patterns and pathways in IPF were extensively investigated, signatures based on transcriptomes, deoxyribonucleic acid (DNA) methylomes, and proteomes were widely used to distinguish IPF from healthy lung tissue and predict IPF prognosis [28].

Analysis of BAL is a useful procedure for differential diagnosis of interstitial lung diseases (ILDs) and for identification of granulomatous lung diseases. Recent studies have identified potentially useful peripheral and BAL biomarkers, including chemokines, cytokines, genes and proteins [29, 30]. Considering low confidence in the estimated differences in the BAL fluid cellular composition of patients with IPF compared with other ILDs, clinical guidelines recommended that BAL fluid cellular analysis should be considered as a diagnostic method for patients who are clinically suspected of having IPF and have an HRCT pattern of probable usual interstitial pneumonia (UIP), indeterminate for UIP, or an alternative diagnosis, not for those with an HRCT pattern of UIP [31, 32]. With respect to prognosis, BAL gene expression profile was well demonstrated to be predictive of mortality in patients with IPF. One interesting study focusing on BAL cell gene expression from three independent IPF cohorts constructed a nine-gene signature for mortality prediction and unrevealed the potential role of airway basal cells in IPF mortality [12]. Other studies based on the previous data and constructed their own predictive models. Another author Li constructed a five-ferroptosis gene signature [33]. One study used ferroptosis-related genes and constructed an 8-ferroptosis-related genes signature to predict prognosis of IPF [34].

In this study, we systematically evaluated the mRNA expression levels of TRIM family members in IPF and healthy samples. Four of DETGs were associated with OS of IPF Patients, which suggested that TRIM genes participated in IPF and harbored a potential predictive value of these genes. The four DETGs (TRIM7, MEFV, TRIM45 and TRIM47), were used to establish a predictive signature. TRIM7, also known as glycogenin-interacting protein 1 (GNIP1), was found to exert protective or detrimental effect in various cancers by different molecular mechanisms [35–37]. MEFV, also known as TRIM20, was found to recognize the inflammasome components and specialize in the suppression of inflammasome and immune activation engendering IL1B/interleukin-1β production [38]. TRIM45 was found to be against in the tumorigenesis of lung cancer by promoting cell apoptosis through activating p38 signal [39]. Moreover, the mRNA expression of TRIM47 in human embryonic lung fibroblast was significantly increased in the IPF group, and TRIM47 over-expression elevated the phosphorylation of Smad2/3 to aggravate fibrosis [40].

Interestingly, members of the TRIM family as E3 ubiquitin ligases were found to critically regulate innate immunity and antiviral response [41]. Studies in individuals with IPF and mouse models strongly support a role for immune dysregulation in promoting the development of pulmonary fibrosis [42]. Recently, there has been interest in the role of tissue-resident memory (TRM) cells in disease. The available data on TRM cells in pulmonary fibrosis are limited, but several important findings have been made. The first was the observation that BAL from IPF patients contains an increased number of TRM CD4+ T cells expressing the integrin CD103. In addition, improved DLCO and survival were observed in IPF patients who had an increased number of circulating resting memory CD4+ T cells [43]. Mast cells were significantly elevated in human IPF lung and positively associated with the number of fibroblast foci; Nintedanib inhibited survival and activation of mast cells and thus provides a novel additional mechanism by which this drug may exert anti-fibrotic effects in patients with IPF [44].

To our knowledge, this study is the first one to investigate one specific gene family signature in IPF, which was more convenient to conduct gene analysis. Considering the significant roles of TRIM family members in various disease processes, our signature consists of only four TRIM genes, which were more concise than other models and have comparable predictive values with other models. However, there are still some limitations in this study. Firstly, the value of signature needs more validation in daily clinical work. Moreover, the signature was constructed to predict prognosis, and not able to predict a patient’s response to medical treatment.

## Conclusions

The TRIM family genes are significant for the prognosis of IPF and our signature could serve as a concise and robust model to predict OS.

## Data Availability

The Data used in the current study was obtained from publicly available repository. The datasets generated or analyzed during the current study are available in the GEO database (GSE70866) repository, https://www.ncbi.nlm.nih.gov/geo/query/acc.cgi?acc=GSE70866.

## Abbreviations

TRIM: Tripartite motif
IPF: Idiopathic pulmonary fibrosis
GEO: Gene expression omnibus
DETGs: Differentially expressed TRIM genes
OS: Overall survival
BAL: Bronchoalveolar lavage
CCD: Coiled-coil domain
HGNC: HUGO Gene Nomenclature Committee
log2FC: Log2FoldChange
FDR: False discovery rate
GSEA: Gene set enrichment analysis
KEGG: Kyoto Encyclopedia of Genes and Genomes
NES: Normalized enrichment score
NK: Natural killer
qRT-PCR: Quantitative real-time PCR
GAP: Gender (G) age (A) lung physiology (P)
FVC: Forced vital capacity
DLCO: Diffusion capacity of the lung for carbon monoxide
VC: Vital capacity
DNA: Deoxyribonucleic acid
ILDs: Interstitial lung diseases
GNIP1: Glycogenin-interacting protein 1
TRM: Tissue-resident memory
